# Olfactory and Gustatory Dysfunctions Following COVID-19 Infection: Factors That Affect Their Duration in Saudi Arabia

**DOI:** 10.7759/cureus.37317

**Published:** 2023-04-09

**Authors:** Yasser B Hennawi, Raneem A Alahmadi, Eithar AlOtaibi, Ashwaq N Alosaimi, Ghada S Tashkandi, Nagham E Saleem, Rahaf I Bukhari, Majed Obaid

**Affiliations:** 1 Faculty of Medicine, Umm Al-Qura University, Makkah, SAU; 2 Faculty of Dentistry, Umm Al-Qura University, Makkah, SAU; 3 Department of Surgery, Umm Al-Qura University, Makkah, SAU; 4 Department of Community Medicine and Pilgrims Healthcare, Umm Al-Qura University, Makkah, SAU

**Keywords:** anosmia, covid-19, factors, duration, post-covid-19, chemosensory dysfunction, loss of taste, ageusia, loss of smell

## Abstract

Introduction: Many people infected with coronavirus disease 2019 (COVID-19) have developed post-COVID-19 symptoms, which are defined as symptoms and signs (e.g., anosmia and ageusia) that persist for more than 12 weeks after getting infected with COVID-19. These symptoms may appear after or during the infection and cannot be explained by any alternative disease. In this study, we aim to investigate the factors that affect the duration of anosmia and ageusia in Saudi Arabia.

Methods: We conducted a nationwide, cross-sectional study using an online survey in Saudi Arabia from 14 February 2022 to 23 July 2022. The electronic survey was distributed using social media platforms, such as Twitter, WhatsApp, and Telegram.

Result: The study enrolled 2497 individuals who were infected with COVID-19. A total of 60.1% of the participants showed symptoms of anosmia, ageusia, or both after getting infected with COVID-19. According to our data, we found that being a female and not having a repeated COVID-19 infection were risk factors (independent predictors) of the long duration of anosmia after COVID-19 recovery (p = <0.05). While being a male patient, a smoker, and being admitted to the ICU were risk factors (independent predictors) of long duration of ageusia after COVID-19 recovery (p = <0.05).

Conclusion: In conclusion, the prevalence of chemosensory dysfunction symptoms, both olfactory and gustatory, after COVID-19 infection among the Saudi population was high. However, several factors can influence their duration, including gender, smoking, and severity of the infection.

## Introduction

The coronavirus disease 2019 (COVID-19) outbreak is caused by severe acute respiratory syndrome coronavirus 2 (SARS-CoV-2), which resulted in a worldwide crisis of a highly infectious disease [1].

The clinical picture of COVID-19 symptoms is a dynamic process in which healthcare workers get a clearer image by observing numerous manifestations affecting different organs. Anosmia (loss of smell) and ageusia (loss of taste) are predictive symptoms and the most common chemosensory dysfunction complaints in post-COVID-19 syndrome. This syndrome is defined by persistent clinical signs and symptoms that appear while or after suffering from COVID-19 infection for more than 12 weeks and cannot be explained by an alternative diagnosis [2,3].

As there are many viruses, such as Epstein-Barr virus, rhinovirus, and para influenza virus, which can lead to smell and taste disorders, different studies have reported that SARS-CoV-2 can also result in chemosensory dysfunctions [4,5]. Hence, several pieces of evidence revealed that olfactory disorder seems to be more prevalent than gustatory dysfunctions [4,6].

The exact pathogenesis behind the mechanism of olfactory and gustatory dysfunctions following the infection remains unclear. However, few studies have reported different hypotheses, in which the virus affects mainly the neurons present in the cerebral cortex and hypothalamus [2,5].

Evidence shows that both anosmia and ageusia are highly prevalent in Saudi Arabia during and after the COVID-19 infection [7,8]. Additionally, patients with anosmia and ageusia may experience a marked decline in quality of life [9]. Therefore, we aimed in this study to investigate the factors affecting the duration of these olfactory and gustatory dysfunctions following the infection.

## Materials and methods

Study design and participants

This descriptive cross-sectional study was conducted among patients with a confirmed diagnosis of COVID-19 by rhino‐pharyngeal swab and patients who presented with symptoms of anosmia and ageusia in the northern, southern, eastern, western, and central regions of Saudi Arabia in 2022. We excluded patients who did not confirm the diagnosis by rhino‐pharyngeal swab.

Ethical consideration and sampling strategy

After structuring the questionnaire using Google Forms (Google, Mountain View, CA), it was publicized to the Saudi community on social media platforms such as WhatsApp, Telegram, and Twitter, and data were collected from 14 February 2022 to 23 July 2022. Ethical approval was obtained from the Biomedical Ethics Committee at Umm Al-Qura University (UQU), College of Medicine, Makkah, KSA, on 14 February 2022 (IRB: HAPO-02-K-012-2022-02-955).

Using Raosoft Sample Size Calculator software (Raosoft, Inc., Seattle, WA) based on the Saudi Arabia population, the minimum sample size to accomplish a precision of 5% with a 95% confidence interval (CI) was obtained as 385 patients [10]. However, our final sample size during data collection included 2497 participants who were infected by COVID-19.

Study tool

The study included 2497 participants. A web-based questionnaire was used. The survey was adapted based on a previously published study [2]. It was formulated in Arabic and English. Furthermore, to ensure clarity and simplicity, a pilot study was conducted. The results of the pilot study were excluded from the final analysis.

The questionnaire was composed of four parts, including consent for participation in the study, demographics data, data about COVID-19 infection, such as COVID-19 severity, vaccination status, and recurrent COVID-19 infection, and the last part collected data on risk factors of the long duration of chemosensory dysfunction.

Statistical analysis

The obtained data were initially gathered in an Excel sheet (Microsoft Corporation, Redmond, WA). Afterward, we used SPSS software version 26 (IBM Corp., Armonk, NY) for data analysis. The chi-squared test (χ2) was applied to qualitative data that were expressed as numbers and percentages to examine the relationship between the variables. The Mann-Whitney and Kruskal-Wallis tests were used to analyze the relationship between the quantitative non-parametric variables that were expressed as mean and standard deviation (mean ± SD). Spearman's test was used for correlation analysis. Multivariate logistic regression analysis was done to determine risk factors of long-duration of anosmia and ageusia after COVID-19 recovery. The odds ratio was calculated at a CI of 95%. A p-value of less than 0.05 was regarded as statistically significant.

## Results

**Figure 1:**
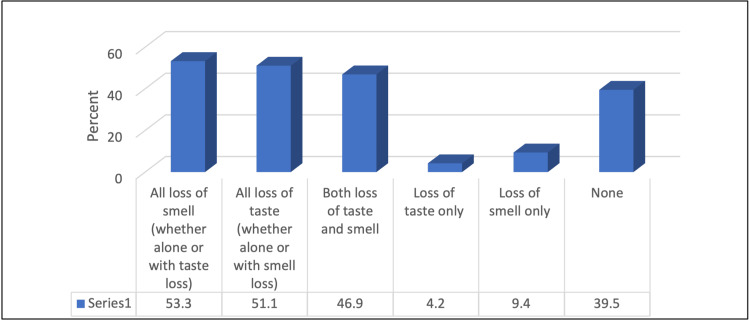
Percentage distribution of the participants according to anosmia and ageusia associated with COVID-19 infection

A total of 4158 individuals participated in the study. We included 2497 participants who confirmed the diagnosis of COVID-19 by rhino‐pharyngeal swab. Among those patients, 53.3% had anosmia and 51.1% had ageusia, as shown in Figure 1. Around 38.4% of the participants were aged ≤ 24 years. In addition, most of the participants were females (67.7%). Their mean BMI was 26.19 ± 6.46 kg/m^2^. The most common blood group was O+ (43.6%). Only 11.5% of participants were smokers, with a mean smoking duration of 10.39 ± 9.79 years and a mean number of daily smoked cigarettes of 11.24 ± 9.36. Only 1% were drinking alcohol. In addition, 34.6% had chronic diseases, and the most common diseases were diabetes mellitus (34.3%) and hypertension (30.9%). Only 18.5% were receiving medications. Furthermore, 15.9% of the study participants were infected with COVID-19 repeatedly, with a mean of 2.09 ± 0.29 times. A total of 76.5% of them were immunized against COVID-19. The majority (29.5%) had two doses with a mean number of doses of 1.62 ± 0.108 doses. A total of 4.6% of patients had chronic rhinosinusitis in the past. Study characteristics are provided in detail in Tables 1, 2.

**Table 1:** Distribution of studied participants according to their age, gender, blood group, smoking, alcohol consumption, chronic diseases, and medication

Variable	No. (%)
Age (years)	
≤24	960 (38.4)
25-40	800 (32)
41-60	691 (27.7)
>60	46 (1.8)
Gender	
Female	1691 (67.7)
Male	806 (32.3)
BMI (kg/m^2^)	26.19 ± 6.46
Blood group	
A+	765 (30.6)
A-	63 (2.5)
B+	328 (13.1)
B-	35 (1.4)
O+	1088 (43.6)
O-	112 (4.5)
AB+	97 (3.9)
AB-	9 (0.4)
Smoker	
Yes	287 (11.5)
No	2210 (88.5)
If a smoker: smoking duration (years)	10.39 ± 9.79
Number of smoked cigarettes (daily)	11.24 ± 9.36
Do you drink alcohol?	
Yes	24 (1)
No	2473 (99)
Chronic diseases	
Yes	864 (34.6)
No	1633 (65.4)
If yes, what disease? (No.: 864)	
Chronic allergy	182 (21)
Psychological disease	51 (5.9)
Obesity	166 (19.2)
Diabetes mellitus	297 (34.3)
Thyroid disorder	117 (13.5)
Hypertension	267 (30.9)
Respiratory disorder	183 (21.1)
Psoriasis	17 (1.9)
Immunological disorder	16 (1.8)
Cardiovascular diseases	66 (7.6)
Are you receiving any chronic medications?	
Yes	463 (18.5)
No	2034 (81.5)

**Table 2:** Distribution of studied participants according to circumstances related to COVID-19 infection

Variable	No. (%)
Have you been infected with COVID-19 repeatedly?	
Yes	398 (15.9)
No	2099 (84.1)
If yes, how many times have you contracted COVID-19?	2.09 ± 0.29
Status of immunization against COVID-19 at the time of COVID-19 infection?	
Not vaccinated	586 (23.5)
Vaccinated	1911 (76.5)
If vaccinated, how many doses? (No.: 1911)	
2 doses	980 (39.5)
3 doses	575 (23)
1 dose	356 (14.3)
Number of doses (mean ± SD)	1.62 ± 0.108
What are the symptoms of COVID-19 that you have?	
Headache	1974 (79)
Fever	1683 (67.4)
Cough	1344 (53.8)
Nausea vomiting	501 (20)
Nose congestion	1484 (59.4)
Sore throat	1558 (62.3)
Runny nose	1466 (58.7)
Dyspnea	686 (27.4)
Body ache	1083 (43.3)
Fatigue	660 (26.4)
Diarrhea	383 (15.3)
Loss of taste	106 (4.2)
Loss of smell	234 (9.4)
What was the first symptom to appear?	
Sore throat	525 (21)
Dyspnea	60 (2.4)
Cough	199 (7.9)
Body aches	24 (0.9)
Fatigue	108 (4.3)
Nose congestion	46 (1.8)
Fever	507 (20.3)
Diarrhea	15 (0.6)
Runny nose	108 (4.3)
Headache	381 (15.2)
Loss of taste	56 (2.2)
Loss of smell	77 (3.1)
How long do COVID-19 symptoms last?	
1-3 days	846 (33.9)
4-6 days	1031 (41.3)
≥7 days	620 (24.8)
Past history	
Nasal fracture	12 (0.5)
Chronic rhinosinusitis	116 (4.6)
Smell alteration before COVID	55 (2.2)
Taste alteration before COVID	34 (1.4)
Malnutrition	53 (2.1)
Surgery or radiotherapy in oral or nasal cavities	16 (0.6)
Antibiotics use	14 (0.6)
Allergic rhinitis	43 (1.7)
Stroke	2 (0.1)
Nasal polyps	10 (0.4)
Multiple sclerosis	1 (0.01)
Head injury	2 (0.1)
Epilepsy	2 (0.1)
Did you need to be hospitalized due to COVID-19?	
Yes	65 (2.6)
No	2432 (97.4)
If the answer is yes, please mention the number of days spent in the hospital	7.22 ± 11.57
Did you need ICU admission?	
Yes	13 (0.5)
No	2484 (99.5)
If yes, for what duration (days)	13.57 ± 2.81
Did you need therapeutic oxygen?	
Yes	245 (9.8)
No	2252 (90.2)
Did the sense of taste change in certain foods only after confirming the infection with COVID-19? (Who lost taste only or both = 1282)	
Yes	413 (32.2)
No	869 (67.8)
If the answer is yes, please mention the foods: (No.: 413)	
All food	330 (79.9)
Fruits	4 (0.9)
Poultry and protein food	19 (4.8)
Salty and spicy	41 (9.9)
Sweets	13 (3.1)
Vegetables	5 (1.2)
Vegetables and fruits	1 (0.2)
How long did the change in the sense of taste toward this type of food last?	34.93 ± 79.49
How long did the complete loss of sense of smell last after recovering from COVID-19?	3.48 ± 1.54
How long did the complete loss of taste last after recovering from COVID-19?	3.17 ± 0.1.38
Have you received any treatment for loss of sense of smell? (1282)	
No treatment	1311 (83.8)
Cortisone	164 (12.7)
Zink	14 (1)
Vitamin C	4 (0.3)
Herbal treatment	28 (2.2)

**Figure 2:**
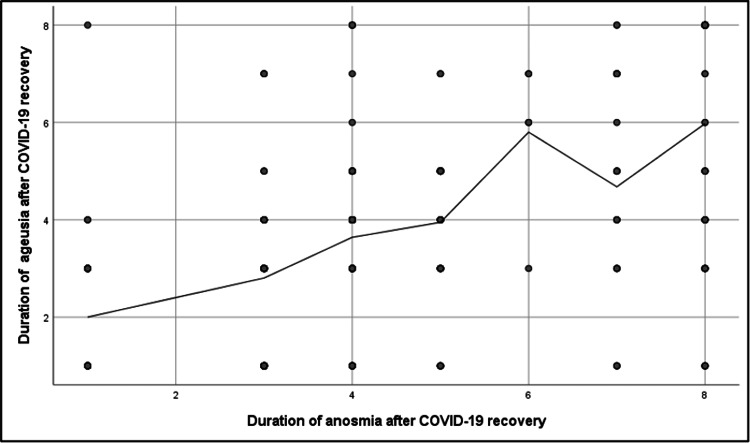
Spearman's test correlation analysis between anosmia and ageusia duration after COVID-19 recovery

A highly significant positive correlation was found between anosmia and ageusia durations after COVID-19 recovery (r = 0.6, p = <0.001) (Figure 2). Regarding the duration of anosmia after COVID-19 recovery, patients aged 41-60 years, females, those without repeated COVID-19 infection, and patients who were not vaccinated against COVID-19 or took only one dose had a significantly higher mean duration of anosmia (p = <0.05). Additionally, patients who had COVID-19 symptoms for ≥ seven days and patients who needed hospitalization or therapeutic oxygen also had a significantly higher mean duration of anosmia (p = <0.05). Considering the duration of ageusia after COVID-19 recovery, male patients, patients who were not vaccinated against COVID-19, and patients who had COVID-19 symptoms for ≥ seven days had a significantly longer mean duration of ageusia (p = <0.05). Furthermore, patients who needed hospitalization or therapeutic oxygen and patients who had head injuries had a significantly longer mean duration of ageusia (p = <0.05) (Tables 3, 4).

**Table 3:** Relationship between duration of anosmia and ageusia and participants' age, gender, blood group, smoking, alcohol consumption, chronic diseases, medication, and past history P-values in bold indicate statistical significance.

Variable	Duration of anosmia after COVID-19 recovery (weeks)	Test (p-value)	Duration of ageusia after COVID-19 recovery (weeks)	Test (p-value)
Age (years)				
≤24	3.36 ± 1.37	3 (0.025)	3.13 ± 1.29	3 (0.668)
25-40	3.54 ± 1.56		3.21 ± 1.47	
41-60	3.58 ± 1.42		3.18 ± 1.4	
>60	3.08 ± 1.38		3.08 ± 1.14	
Gender				
Female	3.61 ± 1.55	5.67 (<0.001)	3.29 ± 1.49	5.01 (<0.001)
Male	3.2 ± 1.17		2.9 ± 1.07	
Blood group				
A+	3.43 ± 1.45	7 (0.368)	3.15 ± 1.35	7 (0.41)
A-	3.13 ± 1.07		2.93 ± 1.21	
B+	3.45 1.35		3.09 ± 1.23	
B-	3.32 ± 1.35		2.91 ± 1.54	
O+	3.52 ± 1.45		3.21 ± 1.49	
O-	3.52 ± 1.35		3.23 ± 1.28	
AB+	3.54 ± 1.12		3.26 ± 1.06	
AB-	3.8 ± 0.38		6.6 ± 0.89	
Smoker				
Yes	3.54 ± 1.51	0.49 (0.618)	3.29 ± 1.44	1.16 (0.245)
No	3.47 ± 1.45		3.15 ± 1.37	
Do you drink alcohol?				
Yes	3.89 ± 2.02	0.75 (0.451)	3.67 ± 1.6	1.31 (0.188)
No	3.47 ± 1.45		3.16 ± 1.8	
Chronic diseases				
Yes	3.47 ± 1.49	0.31 (0.757)	3.12 ± 1.4	1.73 (0.082)
No	3.48 ± 1.44		3.19 ± 1.37	
Do you receive treatment for any of the following chronic diseases?				
Yes	3.37 ± 1.34	1.46 (0.143)	3.13 ± 1.41	0.78 (0.436)
No	3.5 ± 1.48		3.17 ± 1.38	
Past history				
Nasal fracture				
Yes	3.5 ± 0.75	0.08 (0.1934)	1.13 ± 0.25	0.44 (0.659)
No	3.48 ± 1.46		1.03 ± 0.1	
Chronic rhinosinusitis				
Yes	3.35 ± 1.27	1.03 (0.3)	1.05 ± 0.21	1.11 (0.267)
No	3.42 ± 1.3		1.11 ± 0.3	
Smell alteration before COVID-19				
Yes	3.53 ± 1.6	0.57 (0.563)	1.12 ± 0.32	0.76 (0.447)
No	4.38 ± 1.54		1.08 ± 0.21	
Taste alteration before COVID-19				
Yes	3.76 ± 1.7	1.07 (0.281)	1.1 ± 0.3	0.21 (0.826)
No	4.47 ± 1.45		1.03 ± 0.21	
Malnutrition				
Yes	3.64 ± 2.76	0.65 (0.516)	1.06 ± 0.24	0.45 (0.648)
No	3.47 ± 1.44		1.08 ± 0.42	
Surgery or radiotherapy in oral or nasal cavities				
Yes	4.08 ± 1.29	1.46 (0.144)	1.17 ± 0.38	1.06 (0.286)
No	4.37 ± 1.53		1.07 ± 0.23	
Antibiotics use				
Yes	3.25 ± 2.18	0.08 (0.934)	1.02 ± 0.03	0.84 (0.396)
No	3.48 ± 1.45		1.07 ± 0.25	
Allergic rhinitis				
Yes	3.71 ± 1.32	0.14 (0.886)	1.11 ± 0.31	0.48 (0.628)
No	3.47 ± 1.46		1.08 ± 0.27	
Stroke				
Yes	3.14 ± 1.76	0.01 (0.998)	1 ± 0.3	0.01 (0.976)
No	3.38 ± 1.52		1.01 ± 0.4	
Nasal polyps				
Yes	3.5 ± 2.34	0.39 (0.696)	1.17 ± 0.4	0.75 (0.45)
No	3.48 ± 1.6		1 ± 0.03	
Multiple sclerosis				
Yes	3.16 ± 1.2	0.03 (0.869)	1.6 ± 0.76	0.04 (0.667)
No	3.22 ± 1.7		1.01 ± 0.2	
Head injury				
Yes	6 ± 2.82	1.7 (0.089)	1.5 ± 0.7	2.15 (0.03)
No	3.47 ± 1.45		1.03 ± 0.6	
Epilepsy				
Yes	3 ± 1.01	0.51 (0.609)	1 ± 0.01	0.42 (0.672)
No	3.4 ± 1.3		1.03 ± 0.3	

**Table 4:** Distribution of studied participants according to circumstances related to COVID-19 infection P-values in bold indicate statistical significance.

Variable	Duration of anosmia after COVID-19 recovery (weeks)	Test (p-value)	Duration of ageusia after COVID-19 recovery (weeks)	Test (p-value)
Have you been infected with COVID-19 repeatedly?				
No	3.69 ± 1.49	3.17 (0.001)	3.3 ± 1.45	1.61 (0.106)
Yes	3.42 ± 1.44		3.13 ± 1.36	
Status of immunization against COVID-19 at the time of COVID-19 infection?				
Not vaccinated	3.75 ± 1.69	3.57 (<0.001)	3.36 ± 1.52	3.57 (<0.001)
Vaccinated	3.36 ± 1.32		3.08 ± 1.31	
If vaccinated, how many doses? (No.: 1911)				
1 dose	3.75 ± 1.69	2 (0.003)	3.63 ± 1.52	2 (0.556)
2 doses	3.38 ± 1.32		3.18 ± 1.29	
3 doses	3.32 ± 1.22		3.04 ± 1.22	
How long do COVID-19 symptoms last?				
1-3 days	3.3 ± 1.51	2 (<0.001)	3 ± 1.38	1 (0.001)
4-6 days	3.44 ± 1.39		3.14 ± 1.31	
≥7 days	3.69 ± 1 47		3.36 ± 1.46	
Did you need to be hospitalized due to COVID-19?				
Yes	3.78 ± 1.58	2.13 (0.032)	3.5 ± 1.66	1.51 (0.131)
No	3.47 ± 1.45		3.15 ± 1.37	
Did you need ICU admission?				
Yes	3.92 ± 1.78	0.93 (0.35)	3.15 ± 1.37	2.22 (0.026)
No	3.47 ± 1.45		4.42 ± 2.15	
Did you need therapeutic oxygen?				
Yes	3.79 ± 1.56	3.51 (<0.001)	3.51 ± 1.54	3.36 (0.001)
No	3.43 ± 1.43		3.12 ± 1.35	

Table 5 illustrates a significant positive correlation between the duration of anosmia after COVID-19 recovery and patients' age and duration of ageusia after COVID-19 recovery (p = <0.05). At the same time, a significant negative correlation was found between the duration of ageusia after COVID-19 recovery and the number of vaccination doses (p = <0.05) (Table 6).

**Table 5:** Distribution of studied participants according to the severity of COVID-19 infection and associated duration of anosmia P-values in bold indicate statistical significance.

Variable	Duration of anosmia after COVID-19 recovery	
	r	p-value
Age	0.06	0.015
BMI	-0.004	0.881
Smoking duration	-0.04	0.555
Number of smoked cigarettes	0.02	0.882
How many times have you contracted COVID-19?	-0.1	0.065
Number of vaccination doses	-0.07	0.002
Hospital stay (days)	0.1	0.572
ICU duration (days)	0.4	0.428
Duration of ageusia after COVID-19 recovery	0.2	<0.001

**Table 6:** Distribution of studied participants according to the severity of COVID-19 infection and associated duration of ageusia The p-value in bold indicates statistical significance.

Variable	Duration of ageusia after COVID-19 recovery	
	r	p-value
Age	0.02	0.252
BMI	-0.1	0.672
Smoking duration	-0.002	0.975
Number of smoked cigarettes	0.01	0.814
How many times have you contracted COVID-19?	-0.08	0.139
Number of vaccination doses	-0.07	0.002
Hospital stay (days)	0.17	0.342
ICU duration (days)	0.49	0.321

Multivariate logistic regression analysis assessed the risk factors (independent predictors) of the long duration of anosmia and ageusia after COVID-19 recovery among studied patients (Table 7). It was found that being a female and not having a repeated COVID-19 infection were risk factors (independent predictors) of the long duration of anosmia after COVID-19 recovery (p = <0.05). While being a male patient, a smoker, and being admitted to the ICU were risk factors (independent predictors) of long duration of ageusia after COVID-19 recovery (p = <0.05).

**Table 7:** Multivariate logistic regression analysis of risk factors of long duration of anosmia and ageusia after COVID-19 recovery P-values in bold indicate statistical significance.

Variable	Duration of anosmia after COVID-19 recovery			
	B	Wald	p-value	Odds ratio (CI: 95%)
Age (years)	0.03	1.81	0.37	1.13 (0.13-1.3)
Gender	0.09	12.42	<0.001	1.31 (2.31-4.09)
BMI	0.15	1.26	0.334	1.06 (0.94-1.22)
Blood group	0.22	0.56	0.78	1.43 (0.98-1.89)
Smoker	0.36	3.06	0.087	0.66 (0.31-1.98)
Do you drink alcohol?	1.4	5.19	0.189	0.23 (0.17-0.28)
Chronic diseases	0.14	0.78	0.211	0.9 (0.1-1.09)
Are you receiving any chronic medications?	0.1	0.3	0.443	1 (0.12-1.12)
Have you been infected with COVID-19 repeatedly?	0.45	9.7	<0.001	1.9 (2.4-5.09)
Status of immunization against COVID-19 at the time of COVID-19 infection?	0.14	0.11	0.05	0.9 (0.06-1.44)
If vaccinated, how many doses?	0.5	1.22	0.181	0.7 (0.7-1.09)
How long do COVID-19 symptoms last?	0.12	0.23	0.546	1.03 (0.09-1.3)
Did you need to be hospitalized due to COVID-19?	0.23	0.45	0.051	0.9 (0.11-1.34)
Did you need ICU admission?	0.4	0.34	0.675	0.32 (0.13-2.09)
Did you need therapeutic oxygen?	0.08	1.67	0.126	0.61 (0.22-1.08)
Nasal fracture	0.02	0.002	0.968	0.98 (0.38-2.53)
Chronic rhinosinusitis	0.49	1.58	0.209	0.61 (0.28-1.31)
Smell alteration before COVID	0.2	0.12	0.728	0.81 (0.25-2.62)
Taste alteration before COVID	0.6	0.48	0.485	0.54 (1-2.99)
Malnutrition	1	0.04	0.834	1.1 (0.42-2.89)
Surgery or radiotherapy in oral or nasal cavities	0.3	0.14	0.706	1.35 (0.28-6.53)
Antibiotics use	1.11	2.59	0.107	3.06 (0.87-1.93)
Allergic rhinitis	0.54	1.32	0.249	1.71 (0.68-4.31)
Stroke	1.85	0.92	0.571	0.2 (0.31-1.06)
Nasal polyps	0.12	0.95	0.813	0.03 (0.14-10.86)
Multiple sclerosis	0.3	0.15	0.913	0.91 (0.9-0.12)
Head injury	0.65	0.16	0.501	0.14 (0.64-1.053)
Epilepsy	1.13	1.56	0.601	1.09 (0.05-1.13)
	Duration of ageusia after COVID-19 recovery			
Age (years)	0.08	0.13	0.561	1.02 (0.76-1.06)
Gender	0.9	12.06	<0.001	1.9 (2.09-4.03)
BMI	0.12	2.98	0.0.91	0.04 (0.33-1.09)
Blood group	0.14	0.49	0.39	1.22 (0.09-1.9)
Smoker	0.81	3.05	0.001	1.3 (1.6-3.67)
Do you drink alcohol?	0.31	0.04	0.301	0.8 (0.1-1.08)
Chronic diseases	0.15	0.25	0.225	0.13 (0.01-2.09)
Are you receiving any chronic medications?	0.20	0.13	0.513	0.93 (0.13-1.09)
Have you been infected with COVID-19 repeatedly?	0.12	1.9	0.098	0.14 (0.13-0.98)
Status of immunization against COVID-19 at the time of COVID-19 infection?	0.09	0.15	0.112	1.09 (0.49-2.39)
If vaccinated, how many doses?	0.11	1.23	0.77	0.01 (0.94-1.89)
How long do COVID-19 symptoms last?	0.12	0.211	0.708	1.03 (0.89-1.49)
Did you need to be hospitalized due to COVID-19?	0.18	0.34	0.115	0.05 (0.09-1)
Did you need ICU admission?	0.98	2.32	0.016	1.08 (1.13-3.29)
Did you need therapeutic oxygen?	0.12	1.98	0.221	0.17 (0.09-1.1)
Nasal fracture	0.52	0.69	0.405	1.68 (0.49.107)
Chronic rhinosinusitis	1.71	0.32	0.116	0.19 (0.09-1.12)
Smell alteration before COVID	0.01	0.31	0.115	0.01 (0.39-1)
Taste alteration before COVID	1.09	0.1	0.41	0.11 (0.31.11)
Malnutrition	0.45	0.93	0.331	0.15 (0.3-1.31)
Surgery or radiotherapy in oral or nasal cavities	0.5	0.61	0.912	0.30 (0.4-1.05)
Antibiotics use	0.91	0.87	0.304	0.02 (0.11-2.01)
Allergic rhinitis	0.4	0.51	0.201	0.19 (0.13-1.2)
Stroke	0.9	1.03	0.701	1.09 (0.9-2.07)
Nasal polyps	0.41	0.93	0.132	0.5 (0.19-0.9)
Multiple sclerosis	0.51	1.08	0.414	0.9 (0.39-1.07)
Head injury	1.2	0.31	0.611	0.8 (0.38-1.33)
Epilepsy	1.35	0.91	0.667	0.3 (0.11-0.91)

## Discussion

Loss of smell and taste are the most common chemosensory complaints in patients who were infected with COVID-19, and they may present without any nasal symptoms [4]. Our survey-based study investigated 2497 individuals infected with COVID-19 and showed that more than half of them (60.5%) had presented with chemosensory complaints. As there is an increasing number of studies indicating that the most common otolaryngologic symptoms in post-COVID-19 patients are smell and taste dysfunctions [4], therefore, we aimed in this study to investigate the factors that affected these symptoms' duration.

A global systematic review pooled the prevalence of olfactory and gustatory dysfunction among patients who were infected with COVID-19, with percentages of 52.73% and 43.93%, respectively [11]. In comparison to our study, among 2497 patients who recovered from the infection, 53.3% had olfactory dysfunction and 51.1% had gustatory dysfunctions, respectively (Figure 1). These findings are important as they support the association between chemosensory dysfunctions and COVID-19 infection.

The sociodemographic data of a nationwide study showed a significant association between the female sex and the incidence and persistence of anosmia. This aligns with our female participants, who have a significantly higher mean duration of anosmia (p = <0.05). Furthermore, in the mentioned study, females were found to be more susceptible to experiencing ageusia, while this study's data showed that male participants are more prone to have a higher mean duration of ageusia (p = <0.05) [6,12].

Anosmia and ageusia affected the majority of the population in our study aged 24 years and younger (Table 3). The association between age and duration of anosmia after COVID-19 was significantly high (p = 0.025) (Table 3). This was also reported in a previous study, which indicates that younger patients were more susceptible to the infection [6]. On the other hand, the relation between age and duration of ageusia after infection recovery was not as significant (p = 0.668).

Smoking adversely affects the health state of the lungs and the human immune system. Therefore, smokers are more vulnerable to acquiring infectious conditions. Previously published studies revealed that smoking has a highly statistically significant association with anosmia and ageusia [13,14]. Our study reported a low prevalence of smokers in the present study, with a percentage of 11.5%. Despite this small percentage, smokers showed to have a significantly higher mean only in ageusia duration (p = 0.001).

Our results disagree with a recent nationwide cross-sectional study that involves 7520 patients, which shows an association between chronic illnesses and post-COVID-19 symptoms, including taste and smell dysfunctions [5]. In addition, several studies have reported different comorbidities, including diabetes and hypertension, that matter in post-COVID-19 symptoms [4]. Yet, our study revealed that chronic diseases do not have a strong relationship with the duration of anosmia or ageusia after infection recovery. This can be explained by the range difference in age groups participating in the mentioned studies and ours. The majority of the population in Saudi Arabia ranges between 20 and 39 years of age with a percentage of 37.77% [15]. Based on different national studies, the prevalence of diabetes mellitus, hypertension, and respiratory conditions in Saudi Arabia is higher in individuals aged 46 years and older [16-18]. Most of our participants were aged 24 years and younger, which explains the disagreement with previous studies on the relationship between chemosensory dysfunction and their duration after COVID-19 recovery.

Interestingly, patients with mild COVID-19 symptoms and those who were hospitalized were more prone to COVID-19 symptoms [19], and the length of hospital stay and ICU admission can play a significant role in developing these symptoms [2]. Therefore, our data revealed a strong relationship between the duration of ageusia after recovery and ICU admission (p = 0.047). Meanwhile, ICU admission and hospitalization did not have a big impact on the duration of anosmia after COVID-19 infection (p = 0.175 and p = 0.493, respectively).

Limitations

The questionnaire was self-administered, which might lead to self-reporting bias and recall bias. Moreover, due to the nature of this study being cross-sectional and insufficient data collection, more in-depth clinical information from the participants is needed. Therefore, these survey-based findings should be taken with caution.

## Conclusions

In conclusion, chemosensory dysfunction symptoms are important manifestations of COVID-19 diagnosis, especially in the early stages of the infection.

Our study has reported the high prevalence of chemosensory dysfunctions after COVID-19 infection among the Saudi population. Moreover, our study shows that gender, smoking, ICU admission, and first-time infection with COVID-19 do have a significant impact on the duration of these symptoms after COVID-19 infection. Therefore, these findings may help in future research on COVID-19 infection consequences and treatment. Moreover, healthcare professionals should take these data under consideration in suspected or confirmed COVID-19 cases.
